# Trio-Based Low-Pass Genome Sequencing Reveals Characteristics and Significance of Rare Copy Number Variants in Prenatal Diagnosis

**DOI:** 10.3389/fgene.2021.742325

**Published:** 2021-09-20

**Authors:** Matthew Hoi Kin Chau, Jicheng Qian, Zihan Chen, Ying Li, Yu Zheng, Wing Ting Tse, Yvonne K. Kwok, Tak Yeung Leung, Zirui Dong, Kwong Wai Choy

**Affiliations:** ^1^Department of Obstetrics and Gynecology, The Chinese University of Hong Kong, Shatin, Hong Kong, SAR China; ^2^Shenzhen Research Institute, The Chinese University of Hong Kong, Shenzhen, Hong Kong, SAR China; ^3^Hong Kong Hub of Pediatric Excellence, The Chinese University of Hong Kong, Shatin, Hong Kong, SAR China; ^4^The Chinese University of Hong Kong-Baylor College of Medicine Joint Center For Medical Genetics, Shatin, Hong Kong, SAR China

**Keywords:** low-pass genome sequencing, *de novo*, inherited, copy number variants, prenatal diagnosis

## Abstract

**Background:** Low-pass genome sequencing (GS) detects clinically significant copy number variants (CNVs) in prenatal diagnosis. However, detection at improved resolutions leads to an increase in the number of CNVs identified, increasing the difficulty of clinical interpretation and management.

**Methods:** Trio-based low-pass GS was performed in 315 pregnancies undergoing invasive testing. Rare CNVs detected in the fetuses were investigated. The characteristics of rare CNVs were described and compared to curated CNVs in other studies.

**Results:** A total of 603 rare CNVs, namely, 597 constitutional and 6 mosaic CNVs, were detected in 272 fetuses (272/315, 86.3%), providing 1.9 rare CNVs per fetus (603/315). Most CNVs were smaller than 1 Mb (562/603, 93.2%), while 1% (6/603) were mosaic. Forty-six *de novo* (7.6%, 46/603) CNVs were detected in 11.4% (36/315) of the cases. Eighty-four CNVs (74 fetuses, 23.5%) involved disease-causing genes of which the mode of inheritance was crucial for interpretation and assessment of recurrence risk. Overall, 31 pathogenic/likely pathogenic CNVs were detected, among which 25.8% (8/31) were small (<100 kb; *n* = 3) or mosaic CNVs (*n* = 5).

**Conclusion:** We examined the landscape of rare CNVs with parental inheritance assignment and demonstrated that they occur frequently in prenatal diagnosis. This information has clinical implications regarding genetic counseling and consideration for trio-based CNV analysis.

## Introduction

Prenatal genetic diagnosis is routinely performed in high-risk pregnancies to identify fetal genetic abnormalities, including chromosome aneuploidies (such as Trisomy 21) and pathogenic copy number variants (CNVs; such as deletion and duplications). Chromosomal microarray analysis (CMA) is recommended as the first-tier genetic test in the diagnostic evaluation of fetal structural abnormalities by the American College of Obstetricians and Gynecologists (Levy and Wapner, 2018). CMA provides enhanced resolution for the detection of submicroscopic deletions/duplications compared with G-banded chromosome analysis (Leung et al., 2011; Huang et al., 2014; Yang et al., 2017; Chau and Choy, 2021). The spectrum, incidence, and mode of inheritance (*de novo* or inherited) of clinically significant CNVs in prenatal diagnosis by various CMA platforms have been investigated (Chau et al., 2019). In addition, assignment of parental inheritance of CNVs is not only important for clinical interpretation, as rare *de novo* CNVs are more likely to be pathogenic (Asadollahi et al., 2014), but also essential to provide prognostic information and recurrence risk (Huijsdens-van Amsterdam et al., 2018). For instance, the incidence of *de novo* CNVs was 2.9% (14/488) in fetuses with early preterm birth (Wong et al., 2020). However, due to triplication of the experimental cost for trio-based testing (simultaneous), parental inheritance assignment is often performed sequentially, when a candidate variant of interest has been identified in the proband. In a study curating CMA results in 23,865 prenatal cases (Chau et al., 2019), more than 25% of pathogenic CNVs lacked parental inheritance assignment. Thus, comprehensive understanding of rare CNVs with the mode of inheritance is still not well studied in prenatal diagnosis.

Recent studies have demonstrated the feasibility of applying genome sequencing (GS) for CNV detection in prenatal diagnosis (Choy et al., 2019; Zhou et al., 2021), particularly using a low-pass (low-coverage and high-through) approach (Liang et al., 2014; Dong et al., 2016; Wang et al., 2018; Chau et al., 2020; Wang H. et al., 2020). It offers higher resolution of CNV detection (e.g., CNVs < 100 kb in size) and improved sensitivity in detecting low-level mosaic variants. Thus, low-pass GS provides a higher genetic diagnostic yield compared with CMA (Liang et al., 2014; Dong et al., 2016; Wang et al., 2018; Chau et al., 2020; Chaubey et al., 2020; Wang H. et al., 2020). In particular, both reagent costs and experimental repeat rates were lower compared to CMA platforms (Wang H. et al., 2020), enabling its widespread usage in clinical laboratories (Wang et al., 2018; Wang H. et al., 2020). Parental inheritance assignment of CNVs is commonly performed sequentially, after a variant of interest has been identified in the proband. However, recent studies suggested that GS-based CNV detection methods reveals a high number of small CNVs (<100 kb) (Sudmant et al., 2015; Chau et al., 2020; Collins et al., 2020; Wang H. et al., 2020), and it is difficult to determine their clinical significance with a proband-only approach. A sequential approach increases turnaround time; thus, a trio-based approach may be better suited for prenatal testing, especially when pregnancy management and decision-making are often dependent on timely results. As such, the incidence, spectrum, and mode of inheritance of rare CNVs and the proportion of cases requiring parental analysis are important considerations to guide diagnostic approaches (proband-only, sequential approach, or trio-based) by low-pass GS.

Herein, we performed a prospective trio-based study of 315 consecutive prenatal diagnosis cases to study the incidence, landscape, and characteristics of rare CNVs with mode of inheritance assignment by low-pass GS.

## Materials and Methods

### Subject Enrollment, Sample Recruitment, and Preparation

This study was approved by the Joint Chinese University of Hong Kong–New Territories East Cluster Clinical Research Ethics Committee (CREC Ref. No.: 2016.713). From January 2019 to February 2021, pregnant women referred for trio-based prenatal diagnostic testing by low-pass GS at our Prenatal Genetic Diagnosis Center, Department of Obstetrics and Gynecology, The Chinese University of Hong Kong were enrolled. Each participant provided written informed consent. Their primary referral indications included: (1) abnormal ultrasound findings, (2) positive noninvasive prenatal testing, (3) positive Down syndrome screening, (4) advanced maternal age, (5) family/adverse pregnancy history, and (6) others which included ultrasound soft markers only, maternal anxiety, and rare indications. Prenatal samples including chorionic villi samples (CVS), amniotic fluid (AF), or cord blood were collected simultaneously with parental peripheral blood samples.

Genomic DNA from prenatal and parental samples were extracted with DNeasy Blood and Tissue Kit (Cat No./ID: 69506, Qiagen, Hilden, Germany) and were treated with RNase (Qiagen). DNA was subsequently quantified using a Qubit dsDNA HS Assay kit (Invitrogen, Carlsbad, CA, United States). The DNA integrity was assessed by agarose gel electrophoresis. Quantitative fluorescent polymerase chain reaction (qfPCR) was performed using two panels of short-tandem repeat (STR) markers (P1 and XY) located on chromosomes 18, X, and Y for the detection of maternal cell admixture, polyploidy, and confirmation of biological relationships (Choy et al., 2014). G-banded chromosome analysis (karyotyping) was also performed in 205 cases (65.1%).

### Low-Pass Genome Sequencing

Low-pass GS was performed on each sample essentially as previously described (Wang H. et al., 2020). In brief, 50 ng of genomic DNA was digested (200–300 bp) and repaired by fragmentation-end-repair restriction enzyme (MGI tech Co., Ltd., Shenzhen, China). Next, an A-overhang was added for adapter ligation. The DNA fragments underwent seven cycles of PCR. PCR products from each library were subsequently purified with an Agencourt AMPure XP PCR Purification Kit (Beckman Coulter, Brea, CA, United States). The concentration of each library was measured with a Qubit dsDNA HS Assay Kit (Invitrogen). The libraries were mixed with equal molality into each pool (20–24 samples per two lanes) and were sequenced to a minimal of ∼15 million reads per sample (single-end 50 bp) on an MGISeq-2000 platform (MGI, Wuhan, China). The minimal read depth is estimated to be 0.25-fold, which is determined by multiplying the reads (15 million) and the read length (50 bp), divided by the size of human reference genome (3 Gb).

### QC and Data Processing

For each sample, low-quality reads were filtered and single-end reads were aligned to the human reference genome (GRCh37/hg19) using the Burrows–Wheeler Aligner (BWA) (Li and Durbin, 2009) “Aln” and “Samse” alignment modules with the default settings. Uniquely aligned reads were deposited into adjustable sliding windows (50 kb in length with 5-kb increments) and adjustable non-overlapping windows (5 kb). The coverage of each window was calculated by the sum of read amounts after GC correction and population-scale normalization. The genome-wide standard deviation of the copy ratios from all windows except for windows on aneuploid chromosomes was used as a QC measure as previously described, and 0.1 was set as the cutoff (Chau et al., 2020; Wang H. et al., 2020).

### Copy Number Variant Detection and Determination of Parental Inheritance

The detection of homozygous/heterozygous deletions and duplications/triplications was performed by our reported method (Chau et al., 2020; Wang H. et al., 2020). In brief, (1) aneuploidies were detected based on the difference between the average copy ratio for each chromosome compared to the normal copy ratio (expected as 1), where the degree of deviation from normal copy ratios was used to calculate the mosaic level; (2) regions with putative CNVs (at a resolution of 50 kb) were reported, and their precise breakpoint boundaries were determined using our in-house algorithm “Increment-Rate-of-Coverage” (Dong et al., 2016) based on the copy ratios of the adjustable non-overlapping windows; and (3) homozygous or hemizygous deletions were (at a resolution of 10 kb) called if two or more consecutive non-overlapping windows contained extremely low numbers or absence of aligned reads (copy ratio: 0.0–0.1). For mosaic CNV detection, mosaic levels were calculated as previously reported and the minimal mosaic levels of CNV detection were 30% for small CNVs (<2.5 Mb) and 20% for large CNVs (>2.5 Mb) (Chau et al., 2020).

For each CNV, population-based *U*-test, whole-sample-based *t*-test and whole-chromosome-based *t*-test were performed to eliminate false positives and common population-specific polymorphisms. In addition, CNVs with an allele frequency of <1% in our reported datasets (Dong et al., 2016; Chau et al., 2020; Wang H. et al., 2020) of ethnic Chinese fetuses (*n* > 2,000) were defined as rare CNVs for subsequent analyses.

Lastly, the coordinates and the variant type (homozygous/hemizygous/heterozygous deletion or duplication/triplication) of each rare CNV identified in the proband were compared to that of biological parents to determine the mode of inheritance (*de novo* or inherited).

### Clinical Interpretation of Copy Number Variants

Parental inheritance assignment was required for rare CNVs that involved OMIM disease-causing genes, or disease-causing genes due to haploinsufficient/triplosensitivity in peer-reviewed publications, or by ClinGen Dosage Sensitivity Map,^1^ DECIPHER,^2^ or gnomAD.^3^ Rare CNVs with the mode of inheritance were then classified as pathogenic, likely pathogenic (P/LP), variants of uncertain significance (VUS), likely benign, or benign based on the guidelines of the American College of Medical Genetics and Genomics (ACMG) (Riggs et al., 2019) with criteria, methods, and in-house datasets described in our previous study (Dong et al., 2016; Wang H. et al., 2020).

### Verification of Copy Number Variants and the Mode of Inheritance

**FIGURE 1:**
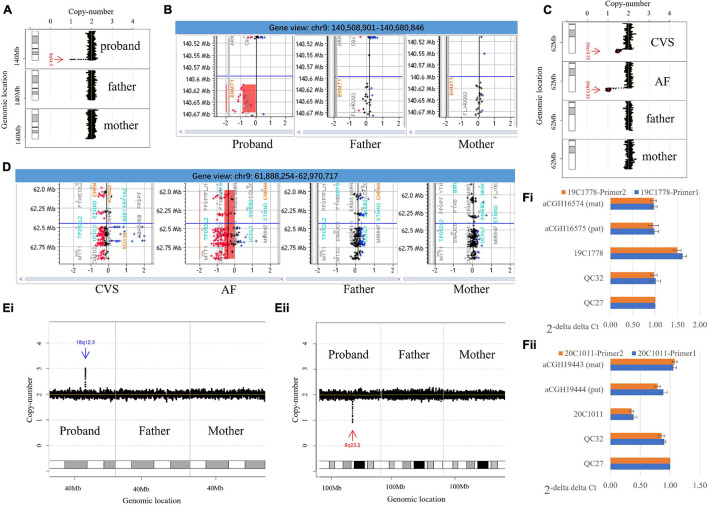
Copy number variants identified by low-pass genome sequencing and the verification. **(A)** Low-pass genome sequencing (GS) identified a 64.7-kb *de novo* heterozygous deletion seq[GRCh37/hg19] del(9)(q34.3)dn chr9:g.140608441_140673160del in case 20C0475. The heterozygous deletion is indicated by a red arrow and was only detected in the proband. **(B)** Chromosomal microarray analysis (CMA) results confirmed a *de novo* heterozygous deletion involving *EHMT1.* **(C)** Low-pass GS detected a 1.0-Mb *de novo* deletion at approximately 50% mosaic level at 20q terminal seq[GRCh37/hg19] del(20)(q13.33)dn chr20:g.61942378_62945038del[0.5] in case 19C3563 on chorionic villi samples (CVS) samples and further identified a constitutional deletion with the same coordinates in an amniotic fluid (AF) sample submitted from the same case. **(D)** CMA analysis confirmed the findings. In panels **(A,C)**, results from low-pass GS: the *X*-axis indicates the copy number of each window (shown as black dot), while the *Y*-axis represents the genomic coordinates. In panels **(B,D)**, results from CMA: the probes in red, black, and blue represent copy number loss, neutral, and gain, respectively. The *X*-axis indicates the log(2) copy ratio of each probe, while the *Y*-axis represents the genomic coordinates. **(Ei)** Low-pass GS identified a *de novo* duplication in 18q12.3 seq[GRCh37] dup(18)(q12.3)dn chr18:g.42995657_43104692dup, which is indicated by a blue arrow. **(Fi)** Quantitative PCR (qPCR) with two independent pairs of primers targeting the region with duplication using QC27 as control. The results indicate a copy number gain in case 19C1778 and copy number neutral in QC32 as well as in each of the parents (mother: aCGH16574; father: aCGH16575). **(Eii)** Low-pass GS identified a *de novo* heterozygous deletion in 8q23.2 seq[GRCh37] del(8)(q23.2)dn chr8:g.111229945_111294607del, which is indicated by a red arrow. **(Fii)** qPCR with two independent pairs of primers targeting the deleted region using QC27 as control. The results indicate a copy number loss in case 20C1011 and copy number neutral in QC32 as well as each of the parents (mother: aCGH19443; father: aCGH19444). In panels **(A,C)**, the *X*-axis indicates the copy number of each window (shown as black dot), while the *Y*-axis represents the genomic coordinates.

Rare *de novo* and P/LP inherited CNVs (Supplementary Tables 1, 2) identified in this study were verified by an orthogonal approach, using either a CMA platform or quantitative PCR (qPCR). For each CNV in query, the 44K Fetal DNA Chip v1.0 (Agilent Technologies, Santa Clara, CA, United States) was assessed for sufficient probe coverage in the region of interest (at least five probes). If this criterion was satisfied, CMA was performed for both the proband and parents simultaneously according to the manufacturer’s protocols. The CNVs were analyzed *via* CytoGenomics software (Leung et al., 2011; Huang et al., 2014; Figure 1A). For CNVs with insufficient probe coverage on our CMA platform, qPCR was performed as previously described (Wang H. et al., 2020). Primers specific to the candidate regions were designed with Primer 3 Web, Primer-Blast (NCBI), and *In Silico* PCR (UCSC) based on the reference genome (GRCh37/hg19). Melting curve analysis was carried out for each pair of primers to ensure specificity of the PCR amplification, and the standard curve method was used to determine PCR efficiency (within a range from 95 to 105%). Each reaction was performed in triplicate in 10 μl of reaction mixtures simultaneously in cases, parents, and control (in-house normal male and female controls) using the SYBR Select Master Mix (Applied Biosystems). The reactions were run on a 7900HT Real-Time PCR System (Applied Biosystems) using the default reaction conditions. The copy numbers in each sample were determined by the ΔΔCt (cycle threshold) method, which compared the difference in Ct of the targeted region with a reference primer pair targeting a universally conserved element in a case against a control. qPCR using two independent pairs of primers (Supplementary Table 3 and Figure 1B) was performed in triplicate to verify each rare *de novo* CNV in each trio.

### Copy Number Variants Curated From ClinVar and in Other Publications

Copy Number Variants curated from ClinVar (Landrum et al., 2014) were downloaded on December 15, 2020, from https://ftp.ncbi.nlm.nih.gov/pub/clinvar/tab_delimited/variant_summary.txt.gz. There were 209,120 variants in total (77,201 copy number gains/duplications and 131,919 copy number losses/deletions). CNVs with conflicting CNV classification were filtered out. There were 4,416 CNVs with sizes no less than 50-kb available for further comparison (GRCh37/hg19).

We also curated CNVs from several published studies on the spectrum of CNVs in prenatal diagnosis for comparison: (1) 428 P/LP CNVs detected in 23,865 prenatal diagnosis cases by CMA, of which clinically relevant CNVs smaller than 10 Mb were included; 25% of the CNVs did not have parental inheritance information (Chau et al., 2019), (2) 51 P/LP CNVs detected in 3,429 cases by low-coverage GS; fetuses with ultrasound anomalies were excluded and only 20% of the CNVs had parental inheritance information (Wang et al., 2018); and (3) 217 CNVs (seven P/LP) in 111 cases by trio-based high read-depth GS (Zhou et al., 2021), in which parental inheritance was not provided for CNVs smaller than 100 kb.

### Statistical Analysis

The incidence of CNVs stratified by clinical classification, mode of heritance, and referral indication for invasive testing is shown as proportions with 95% confidence intervals calculated with the Wilson score method without continuity correction. In addition, Kruskal–Wallis rank-sum test was performed to compare the CNV parameters, including the type of aberration, the size, constitutional/mosaicism, and the mode of inheritance in different studies. Lastly, chi-square test or Fisher exact test was performed to compare the incidence of small CNVs between studies. All statistical analyses were performed with the statistical software package SPSS 25.0 (IBM SPSS Statistics for Windows, version 25.0; IBM Corp., Armonk, NY, United States).

## Results

From January 2019 to February 2021, 315 women referred for trio-based prenatal genetic diagnosis at our clinical laboratory were enrolled. There were 54 CVS, 257 AF samples, and 4 fetal cord blood samples. Parental peripheral blood samples were available for all cases. Demographic information including maternal and paternal age, and the gestational week are shown in Supplementary Table 1. After exclusion of maternal cell admixture by qfPCR, all cases were subjected to low-pass GS for CNV analysis. An average of 18 million reads were generated per case, which was equivalent to 0.3-fold. Overall, trio-based low-pass GS provided a 12.4% (39/315) diagnostic yield among the 315 cases (Table 1).

**TABLE 1:** Diagnostic yield in cases with different referral indications.

Clinical indication	Cases enrolled	Cases with diagnosis	Diagnostic yield (%)	[95% CI]	Cases with pathogenic findings (inherited or *de novo*)*	Number of Dup/del	Cases #
Abnormal ultrasound	165	15	9.09%	[5.35, 14.81]	Cases with inherited P/LP CNVs	Del: 3	4(0.02)
						Dup: 1	
					Cases with *de novo* P/LP CNVs	Del: 3	7(0.04)
						Dup: 4	
					Cases with aneuploidies	Del: 1	4(0.02)
						Dup: 3	
Non-invasive prenatal screening - high risk	70	19	27.14%	[17.52, 39.30]	Cases with inherited P/LP CNVs	Del: 1	1(0.01)
						Dup: 0	
					Cases with *de novo* P/LP CNVs	Del: 8	9(0.13)
						Dup: 1	
					Cases with aneuploidies	Del: 2	9(0.13)
						Dup: 7	
1st/2nd Trimester aneuploidy screening high risk (DSS)	16	3	18.75%	[4.97, 46.31]	Cases with inherited P/LP CNVs	Del: 1	1(0.06)
						Dup: 0	
					Cases with *de novo* P/LP CNVs	Del: 2	2(0.13)
						Dup: 0	
					Cases with aneuploidies		0(0)
Advanced maternal age	11	0	0.00%	[0, 32.14]	-		−
Family history	31	2	6.45%	[1.12, 22.84]	Cases with inherited P/LP CNVs		0(0)
					Cases with *de novo* P/LP CNVs	Del: 1#	2(0.06)
						Dup: 2#	
					Cases with aneuploidies		0(0)
Others	22	0	0.00%	[0, 18.5]	Cases with inherited P/LP CNVs		0(0)
					Cases with *de novo* P/LP CNVs		0(0)
					Cases with aneuploidies		0(0)
Total	315	39	12.38%	[9.05, 16.65]	-		39(0.12)

*P/LP refers to pathogenic or likely pathogenic.

*^#^Each digit in the bracket refers to the incidence over the sample enrolled in each subgroup, #20C2527 with duplication and deletion at the same time: seq[GRCh37] dup(Y)(p11.31q11.221)dn chrY:g.2649473_19567688dup, seq[GRCh37] del(Y)(q11.221q12)dn chrY:g.19567689_59033394del.*

### Rare Copy Number Variants Identified in Trio-Based Analysis

**FIGURE 2:**
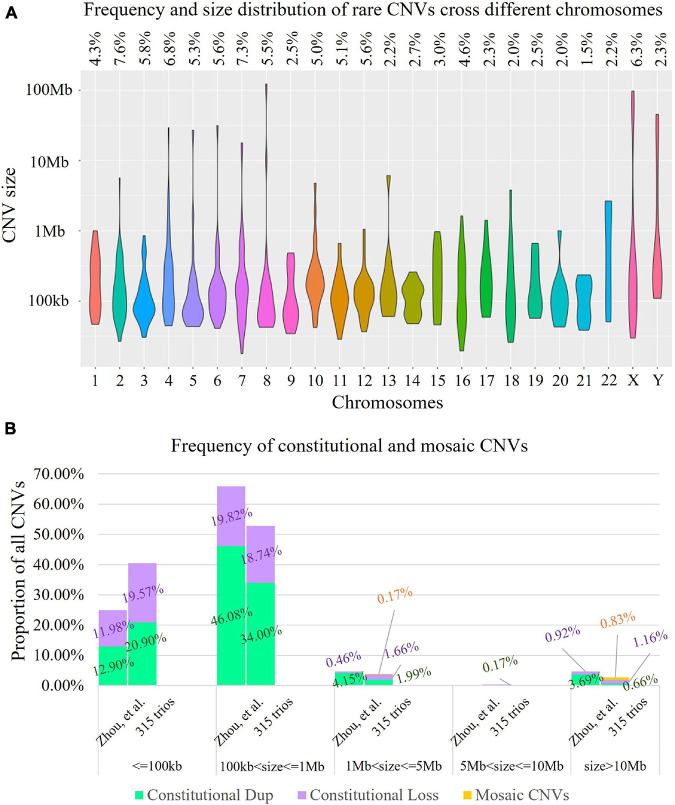
Landscape of rare copy number variants (CNVs). **(A)** Distribution of 603 rare CNVs identified cross different chromosomes (violin plot). The *X*-axis presents different chromosomes, while the *Y*-axis indicates the number of rare CNVs identified (in log10 format). The frequency of rare CNVs in each chromosome is provided in the top panel. **(B)** Comparison of the rare CNVs identified in our study and in a trio-based high read-depth genome sequencing study (Zhou et al., 2021; *n* = 111). In each bar, each segment in green, purple, and yellow indicates the percentage of constitutional duplications (digits in dark green), constitutional deletions (digits shown in purple), and mosaic CNVs (digits in orange) identified. The results indicated that the size distributions were significantly different (Kruskal–Wallis rank sum test: *p* = 0.0054).

Low-pass GS identified 14 constitutional or mosaic aneuploidies in 13 cases (4.1%, 13/315, Supplementary Materials). CNV analysis revealed 603 rare CNVs (>50 kb, homozygous/hemizygous deletion > 10 kb) including 597 constitutional and six mosaic CNVs in 272 fetuses (272/315, 86.3%, Figure 2A), providing roughly 1.9 rare CNVs per case (603/315). On average, 8.84 RefSeq genes were involved in each rare CNV. The majority of rare CNVs were smaller than 1 Mb (562/603, 93.2%), while the six mosaic CNVs were all larger than 1 Mb (Figure 2A). We further compared the size distribution of CNVs to those reported by Zhou et al. (2021) in a trio-based high read-depth GS study utilizing an independent algorithm (*n* = 111). The results indicated the size distributions were significantly different (Kruskal–Wallis rank sum test: *p* = 0.0054, Figure 2B). The large mosaic CNVs reported and the large proportion of small CNVs (<100 kb) in our study may explain the differences in size distribution (Zhou et al., 2021).

**FIGURE 3:**
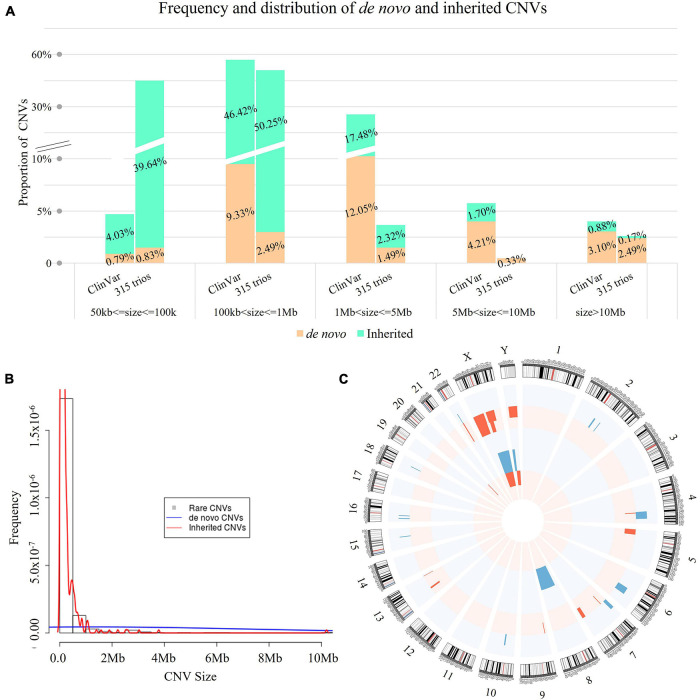
Spectrum of rare CNVs with mode of inheritance. **(A)** Comparison of the rare CNVs identified in our study and in ClinVar (*n* = 4,416) with mode of inheritance. In each bar, each digit in red indicates the percentage of inherited CNVs (cyan bar), while each digit in black represents the percentage of *de novo* CNVs (tan bar). The size distribution of the 603 rare CNVs showed significant difference compared with CNVs curated in ClinVar (*n* = 4,416, Kruskal–Wallis rank sum test: *p* < 0.0001), but not for *de novo* CNVs Kruskal–Wallis rank sum test: *p* = 0.785). **(B)** Histogram of rare CNVs. The density lines in red and blue reflect the size distribution of inherited and *de novo* CNVs, respectively. The size distribution was significantly different between *de novo* and inherited CNVs (Kruskal–Wallis rank sum test: *p* < 0.0001). **(C)** Distribution of *de novo* CNVs identified in our study. Blue bars represent copy number gains and red bars represent copy number losses encompassing the chromosomal bands. The height represents the frequency of the pathogenic copy number variants. The outer circle indicates the distribution of mosaic *de novo* CNVs, while the inner circle presents the distribution of constitutional *de novo* CNVs.

The size distribution of the 603 rare CNVs also showed significant difference compared with CNVs curated in ClinVar (*n* = 4,416, Kruskal–Wallis rank sum test: *p* < 0.0001, Figure 3A). Although our study shared a similar proportion of CNVs ranging from 100 kb to 1 Mb in size (52.74 vs. 55.75%, Chi-square test, *p* = 0.1631), the percentage of small CNVs (from 50 to 100 kb) in our study was significantly higher that of ClinVar (40.47 vs. 4.82%), with over eightfold increase (Chi-square test, *p* < 0.0001).

### Mode of Inheritance

Of the 603 rare CNVs, 46 were *de novo* (in 36 cases, 11.4%, 36/315, Supplementary Table 2) and 557 were inherited (in 248 cases, 78.7%, Figure 3A). The size distribution was significantly different between *de novo* and inherited CNVs (Kruskal–Wallis rank sum test: *p* < 0.0001, Figure 3B). The majority of small CNVs (50–100 kb) in our study were inherited (239/244, 97.95%). In comparison, *de novo* CNVs were larger in size compared with inherited CNVs. They also involved significantly more RefSeq genes (Supplementary Figure 1).

Among the *de novo* CNVs, 40 were constitutional and six were mosaic (Figure 3C), providing a frequency of 0.15 *de novo* CNVs per case (46/315). On average, there were 92.3 RefSeq genes involved in each *de novo* CNV (median: 25 genes). All *de novo* CNVs were validated by CMA or qPCR (see “Materials and Methods” and Figure 1).

There was no significant difference between the size distributions of *de novo* CNVs between our study and ClinVar (Kruskal–Wallis rank sum test: *p* = 0.785, Figure 3A). However, the proportion of small *de novo* CNVs (50–100 kb) was significantly higher than that curated in ClinVar (10.9 vs. 2.7%, Chi-square test: *p* = 0.0013). In addition, there were no significant differences between parental age and the incidence of *de novo* CNVs (Supplementary Figure 2).

### Rare Copy Number Variant Classification and Trio-Based Analysis

**FIGURE 4:**
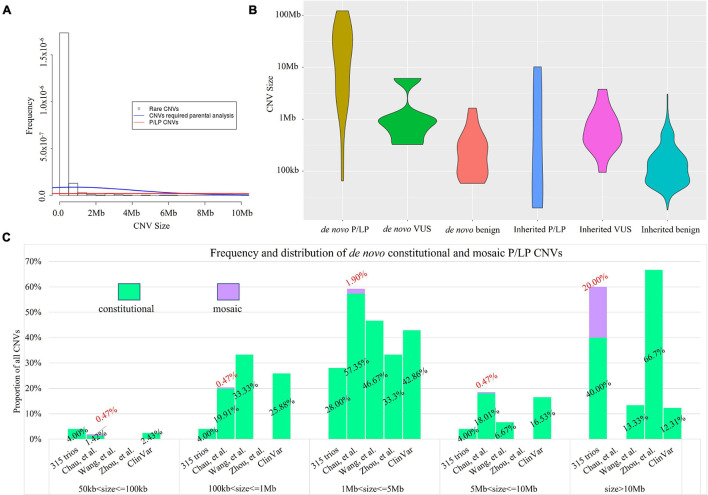
Size distribution of rare CNVs with clinical classification. **(A)** Histogram of rare CNVs. The density lines in red and blue reflect the size distribution of 84 rare CNVs requiring parental analysis and 31 P/LP CNVs, respectively. The median size of the 84 rare CNVs was 725 kb vs. 126 kb of all 603 rare CNVs (Kruskal–Wallis rank sum test: *p* < 0.0001). **(B)** Size distribution of *de novo* and inherited CNVs and the classification. **(C)** Comparison of the size distribution of pathogenic or likely pathogenic CNVs identified in our study with ones reported in a trio-based high read-depth genome sequencing study (Zhou et al., 2021; *n* = 111), the CMA study with largest number of prenatal cases (*n* = 23,865; Chau et al., 2019), and a study with 3,429 prenatal cases by low-coverage GS (Wang et al., 2018). Bars in light green and purple indicate the percentage of CNVs identified in constitutional and mosaic form, respectively.

In this study, we also aimed to determine the percentage of cases with rare CNVs requiring information of parental assignment after proband-only interpretation, which is critical for genetic counseling and consideration for trio-based CNV analysis. We then classified the clinical significance of 603 rare CNVs identified in fetuses following the ACMG guidelines (Riggs et al., 2020). There were 84 rare CNVs in 74 cases that involved disease-causing genes, of which the mode of inheritance was important for the clinical interpretation and estimation of recurrence risk (23.5%, 74/315, see “Materials and Methods” and Supplementary Table 3). The 84 CNVs had a different size distribution compared with the overall 603 rare CNVs (median size: 725 vs. 126 kb, Kruskal–Wallis rank sum test: *p* < 0.0001, Figure 4A). In light of parental inheritance assignment, 31 CNVs (in 26 cases) were classified as P/LP CNVs (Supplementary Table 3), 18 as VUS (in 18 cases), and 35 as benign CNVs. Among the 31 P/LP CNVs (in 26 cases), 25 were *de novo* CNVs, and 6 were inherited. Low-pass GS provided a diagnostic yield of 8.2% (26/315, Table 1). In addition, among the 18 VUS, 5 were *de novo* and 13 were inherited. The incidence of VUS (18/315, 5.7%) was not significantly different from our previous prospective study that performed parental inheritance assignment in a sequential approach (53/1,023, 5.2%, Chi-square test, *p* = 0.7119). Overall, the 31 P/LP CNVs also had significant differences in size distributions compared with 84 CNVs requiring parental analysis (Kruskal–Wallis rank sum test: *p* = 0.0067, Figure 4A). Among different classifications, *de novo* CNVs tended to be larger in size compared with inherited CNVs, particularly P/LP CNVs (Figure 4B).

There were five cases with 22q11.2 deletion syndrome: four cases occurred *de novo*, while one case was maternally inherited. Although all five deletions were classified as P/LP CNVs, their recurrence risks would be different (McDonald-McGinn and Zackai, 2008).

### Clinical Interpretation of Copy Number Variants Based on the Mode of Inheritance

*De novo* CNVs in our cohort are more likely to be classified as P/LP than inherited CNVs (54.35% [25/46] vs. 1.58% [6/567], Chi-square test, *p* < 0.0001). All mosaic CNVs were classified as P/LP CNVs except the 20q13.33 deletion (VUS, Supplementary Table 3). Case 19C3563 was referred for cardiomegaly with abnormal tricuspid valve and abnormal ± pulmonary valve at 15 + 4 gestational weeks. A *de novo* 1.0-Mb deletion of approximately 50% mosaic level was detected, seq[GRCh37/hg19] del(20)(q13.33)dn chr20:g.61942378_62945038del[0.5], and further confirmed by CMA (Figures 1C,D). The gene *KCNQ2* was involved, the haploinsufficiency of which causes neonatal seizures (Heron et al., 2007) and encephalopathy (Spagnoli et al., 2018). The deletion was classified as VUS. To exclude the possibility of confined placental mosaicism, low-pass GS was further performed on the AF sample collected at a later gestational week and revealed a constitutional 20q deletion, further confirmed by CMA (Figures 1C,D). After genetic counseling, the couples opted for termination of pregnancy.

Among all P/LP CNVs, three were smaller than 100 kb. Two cases had Southeast Asian (SEA) type homozygous deletions resulting in α-thalassemia major (19.3-kb deletions due to biparental inheritance), while another case had a *de novo* pathogenic deletion. Case 20C0475 was referred for invasive testing at 16 + 2 gestational weeks due to high-risk Down syndrome screening results (risk at 1:2) and advanced maternal age. Low-pass GS detected a 64.7-kb *de novo* heterozygous deletion seq[GRCh37/hg19] del(9)(q34.3)dn chr9:g.140608441_140673160del involving the exons 3–12 of *EHMT1*, which was confirmed by CMA (Figures 1A,B). Haploinsufficiency of *EHMT1* is known to cause Kleefstra syndrome 1 in an autosomal dominant manner (OMIM #610253). The deletion was classified as pathogenic, and the pregnancy was terminated after genetic counseling. Overall, the most common P/LP CNV identified was recurrent 22q11.2 microdeletion associated with DiGeorge syndrome (*n* = 5), while the other cases had isolated CNVs.

To further investigate whether the size distribution of P/LP CNVs in our cohort was different from previously reported studies, we further curated the CNVs reported in three prenatal studies: 23,865 cases by CMA (Chau et al., 2019), 3,429 cases by low-coverage GS (Wang et al., 2018), and 111 cases by high read-depth GS (Zhou et al., 2021) (see “Materials and Methods”). Parental confirmation was performed in a sequential manner in the first two studies. The size distributions among P/LP CNVs in all studies were significantly different (Kruskal–Wallis rank sum test: *p* < 0.0001). In addition, the size distributions of *de novo* P/LP CNVs in all studies were also significantly different (Kruskal–Wallis rank sum test: *p* < 0.0001, Figure 4C). Particularly, the sizes of all P/LP CNVs and all *de novo* P/LP CNVs in our study were both significantly different from the ones curated in ClinVar (Kruskal–Wallis rank sum test: *p* = 0.0002 and *p* < 0.0001, respectively). In addition, both of them were also significantly different from the ones reported by the study with 3,429 cases by low-coverage GS (Wang et al., 2018) (Kruskal–Wallis rank sum test: *p* = 0.0007 and *p* = 0.0013, respectively). It could be explained by the presence of *de novo* or inherited small P/LP CNVs (<100 kb) and mosaic P/LP CNVs in our study, which accounted for 25.8% of the P/LP CNVs (8/31).

### Incidence of Rare Copy Number Variants in Subgroups With Different Primary Referral Indications

In addition, we further calculated the frequency of rare CNVs and *de novo* CNVs based on the primary referral indications (Table 2). Subgroups of cases with abnormal ultrasound findings and cases with high risk of non-invasive prenatal testing were the two groups with the highest number of cases enrolled (165 vs. 70), and they shared similar incidences of rare CNVs (Table 2). However, the incidence of cases with P/LP *de novo* CNVs in high-risk cases from non-invasive testing (9/70, 12.9%) was higher than cases referred with ultrasound anomalies (4.2%, 7/165, Table 2). For the incidences of cases with rare CNVs with small size (<100 kb) or mosaic CNVs, cases with *de novo* small CNVs or mosaic CNVs, and cases with rare CNVs requiring parental analysis, all were similar between these two subgroups (Table 2).

**TABLE 2:** Incidence and classification of rare copy number variants (CNVs) in cases with different referral indications.

Clinical indication	Cases enrolled	Rare CNVs		*De novo* CNVs		Rare CNVs less than 100 kb or in mosaicism		*De novo* CNVs less than 100 kb or in mosaicism		Rare CNVs required parental analysis	
						
		Cases	Number	Cases	Number	Cases	Number	Cases	Number	Cases	Number
Abnormal ultrasound	165	141 (0.85)	304 (1.84)	17 (0.1)	21 (0.12)	89 (0.53)	127 (0.76)	5 (0.03)	5 (0.03)	41 (0.24)	47 (0.28)
Non-invasive prenatal screening – high risk	70	64 (0.91)	145 (2.07)	13 (0.18)	17 (0.24)	40 (0.57)	59 (0.84)	2 (0.02)	2 (0.02)	18 (0.25)	21 (0.3)
1st/2nd Trimester aneuploidy screening high risk (DSS)	16	15 (0.93)	31 (1.93)	3 (0.18)	3 (0.18)	9 (0.56)	12 (0.75)	1 (0.06)	1 (0.06)	6 (0.37)	6 (0.37)
Advanced maternal age	11	9 (0.81)	13 (1.18)	0 (0)	0 (0)	3 (0.27)	4 (0.36)	0 (0)	0 (0)	0 (0)	0 (0)
Family history	31	26 (0.83)	61 (1.96)	2 (0.06)	3 (0.09)	18 (0.58)	28 (0.9)	1 (0.03)	2 (0.06)	6 (0.19)	7 (0.22)
Others	22	17 (0.77)	49 (2.22)	1 (0.04)	2 (0.09)	11 (0.5)	20 (0.9)	1 (0.04)	1 (0.04)	3 (0.13)	3 (0.13)
Total	315	272 (0.86)	603 (1.91)	36 (0.11)	46 (0.14)	170 (0.53)	250 (0.79)	10 (0.03)	11 (0.03)	74 (0.23)	84 (0.26)

*Each digit in the bracket refers to the incidence over the sample enrolled in each subgroup.*

## Discussion

This is a prospective study of trio-based low-pass GS in prenatal diagnosis, providing the landscape of rare CNVs and the mode of inheritance. Among the 315 fetuses, CNV analysis revealed 603 rare CNVs, namely, 597 constitutional and 6 mosaic CNVs in 272 fetuses (272/315, 86.3%). On average, 1.9 rare CNVs were detected per fetus (603/315). In a previous study on rare CNVs, the array-based method reported a frequency of 0.59 rare CNVs per case (Ruderfer et al., 2016). The average 1.9 rare CNVs identified per fetus in prenatal diagnosis is in line with expectations as GS provides improved genome coverage compared to CMA, albeit at a low-pass/low-coverage setting, shown by our previous studies (Chau et al., 2020; Wang H. et al., 2020).

The majority of CNVs detected in our study were smaller than 1 Mb (562/603, 93.2%), while 1% (6/603) were mosaic. Among all 603 rare CNVs, 46 were *de novo* (7.6%, 46/603), which were detected in 11.4% (36/315) of cases. Overall, 12.4% (39/315 vs. 13.5%, 138/1,023) of cases had pathogenic findings (aneuploidies and/or P/LP CNVs) and 5.7% (18/315 vs. 5.2%, 53/1,023) of cases had VUS, both of which were consistent with our previous study where parental inheritance assignment was performed in a sequential manner (Wang H. et al., 2020). Performing trio-based low-pass GS simultaneously or sequentially do not affect the overall diagnostic yield. However, a sequential approach would increase the turnaround time of testing. In addition, the percentage of cases with rare CNVs requiring information of parental assignment after proband-only interpretation based on ACMG guidelines was 23.5 (74/215, 84 CNVs in 74 fetuses). It would provide potential clinical implications regarding genetic counseling and consideration for trio-based CNV analysis. Nonetheless, for pregnancy management and decision-making that are highly dependent on timely test results, trio-based approach may be recommended.

Among the 315 cases, 603 rare CNVs (allele frequency < 1% in our curated reference dataset of Chinese fetuses (Dong et al., 2016; Chau et al., 2020; Wang H. et al., 2020): *n* > 2,000) were detected, providing an incidence of 1.9 rare CNVs per case (603/315). Of these variants, 40.5% (244/603) were smaller than 100 kb. ClinVar is a database that archives reports of relationships among human genomic variants and phenotypes, with supporting evidence. However, a significant proportion of CNVs submitted to ClinVar was identified by the CMA platform. The differences in size distribution of CNVs between our study and ClinVar may be caused by platform differences. In particular, the percentage of small CNVs (from 50 to 100 kb) in our study was significantly higher than the one curated in ClinVar (40.5 vs. 4.8%) with an over eightfold increase (Chi-square test, *p* < 0.0001). In addition, the percentage of small (50–100 kb) *de novo* CNVs (5/46, 10.9%) was still significantly higher than curated in ClinVar (2.67%, 35/1302) (Chi-square test, *p* = 0.0012). This illustrates a deficiency of rare and small CNVs curated in ClinVar, which would be helpful for laboratory reference in CNV interpretation. Gradual deposition of rare and small CNVs identified by GS would benefit and facilitate prenatal diagnosis of clinically relevant CNVs. Our study not only found P/LP CNVs smaller than 100 kb (*de novo* or inherited), accounting for 9.67% of all detected P/LP CNVs (3/31), but also provided evidence that *de novo* mosaic P/LP CNVs contributed to a significant proportion of pathogenic findings (16.1%, 5/31). Both types of CNVs were not reported in a study with 3,429 prenatal cases by low-coverage GS (Wang et al., 2018). The possible reasons might include the exclusion of cases with ultrasound anomalies and limited resolution of their analysis pipeline (100 kb) (Wang et al., 2018). Significant differences in CNV size distributions between our study and previous studies with different methods (Wang et al., 2018; Zhou et al., 2021) was observed, which might be caused by the different analysis pipelines used. Our findings emphasize the important clinical implication of small CNVs and mosaic CNVs in prenatal diagnosis and warrants a CNV detection method sensitive to small and mosaic variants.

We provided the size distributions of rare CNVs, CNVs requiring parental analysis, and P/LP CNVs. The high abundance of small CNVs was largely contributed by inherited CNVs; clinical interpretation and estimation of recurrence risk largely relied on the mode of inheritance. Parental mode of inheritance assignment was important in nearly a quarter of cases. Recently, there are publications showing the performance of their in-house CNV detection methods using low-pass GS data (Wang et al., 2018; Wang et al., 2020); however, the software/pipelines are not publicly available. As our study aimed to investigate the spectrum and characteristics of rare CNVs, a fair comparison of different methods using low-coverage/low-pass GS for CNV study including pros and cons is warranted in a future study.

A major strength of this study includes the prospective study of 315 prenatal cases with a variety of different clinical indications undergoing invasive prenatal diagnosis. The rare CNV findings represent the spectrum and incidence of *de novo* and inherited CNVs identified among prenatal testing by low-pass GS. Furthermore, our analysis provided a view of rare CNVs by low-pass GS in prenatal diagnosis.

Limitations include (1) limited sample size (*n* = 315) and (2) limited CNV detection resolution (50 kb, homozygous/hemizygous deletion: 10 kb): although our study provided an enhanced resolution compared with the reported studies by GS (Wang et al., 2018) and CMA (Chau et al., 2019), the spectrum and incidence of smaller CNVs (<50 kb) are still not well studied. There are large amounts of small CNVs in human genomes (Collins et al., 2020); trio-based GS analyses using higher read-depths (increased resolution) and larger sample sizes are warranted in future studies. In addition, read-depth-based CNV analysis is unable to assemble derivative chromosomes or identify the genomic locations and orientations of copy number gains. Paired-end sequencing approaches (Talkowski et al., 2012; Dong et al., 2018, 2019, 2021) to further delineate the locations and the breakpoint junctions of CNVs may provide a more comprehensive understanding of prenatally detected CNVs. Particularly, apparently *de novo* deletions or duplications might be caused by balanced rearrangements (such as insertions) in the parents (Nowakowska et al., 2012). Low-pass GS does not detect single-nucleotide variants (SNVs) and small insertions/deletions (indels) that can also be pathogenic in the prenatal context. Early studies have revealed promising diagnostic utility of prenatal ES for the detection of pathogenic SNVs and indels in fetuses with structural abnormalities. Further studies are warranted to examine the clinical utility of prenatal ES to guide its clinical implementation. Lastly, future studies on *de novo* variants in prenatal diagnosis may be extended to the investigation of SNVs/indels (Lord et al., 2019; Petrovski et al., 2019).

## Data Availability Statement

The datasets presented in this study can be found in online repositories. The names of the repository/repositories and accession number(s) can be found in the article/Supplementary Material.

## Ethics Statement

The studies involving human participants were reviewed and approved by Joint Chinese University of Hong Kong-New Territories East Cluster Clinical Research Ethics Committee. The patients/participants provided their written informed consent to participate in this study.

## Author Contributions

MC, YK, TL, ZD, and KC designed the study. WT and TL collected the samples and followed up. JQ, ZC, MC, and ZD performed the analysis and data interpretation. JQ, YL, and YZ conducted the validation. MC, JQ, ZD, and KC wrote the manuscript. All authors contributed to the article and approved the submitted version.

## Conflict of Interest

The authors declare that the research was conducted in the absence of any commercial or financial relationships that could be construed as a potential conflict of interest.

## Publisher’s Note

All claims expressed in this article are solely those of the authors and do not necessarily represent those of their affiliated organizations, or those of the publisher, the editors and the reviewers. Any product that may be evaluated in this article, or claim that may be made by its manufacturer, is not guaranteed or endorsed by the publisher.

## Footnotes

1
https://dosage.clinicalgenome.org/

2
https://www.deciphergenomics.org/

3
https://gnomad.broadinstitute.org/
