# Supplementary material for: Trio-Based Low-Pass Genome Sequencing Reveals Characteristics and Significance of Rare Copy Number Variants in Prenatal Diagnosis
Source: Front Genet. 2021 Sep 20;12:742325. doi: 10.3389/fgene.2021.742325 (PMC8488434; doi:10.3389/fgene.2021.742325)
Supplement: Supplementary file 1 [file Data_Sheet_1.docx]

**Supplementary Materials**

**Chromosomal aneuploidies identified**

In this cohort, 14 constitutional or mosaic aneuploidies was reported in 13 cases (4.1%, 13/315, **Figure 2A** and **Supplementary** **Table S5**), all of which occurred in *de novo*. Among them, five were mosaic aneuploidies involving chromosomes 7, 10, 18, X and Y, while the remaining cases had constitutional common aneuploidies (such as Trisomy X, 21 and Turner syndrome). For instance, case 19C1686 was referred for CVS at 13+5 gestational weeks, and the referral indications included diffuse cystic placenta and placental thickness 2.39 cm. Low-pass GS performed on the uncultured CVS identified a mosaic Trisomy 7 and a mosaic Trisomy 10, both of which were at approximately 7% mosaic level. G-banded chromosome analysis indicated trisomies 7 and 10 came from the same cell line, however, with mosaic level over 95% (48,XY,+7,+10[29]/46,XY[1]). We further conducted low-pass GS with DNA extracted from the cultured cells. The result indicated a mosaic Trisomy 7 and a mosaic Trisomy 10, both of which had approximately 85% mosaic level, similar to that reported by karyotyping. The discrepancy of the mosaic percentages in the direct assessment of DNA extracted from CVS and the cultured CVS was likely due to different cell origins between the direct (uncultured) analysis (predominantly cytotrophoblasts) and cultured cells (mesenchymal core of villi)^15^. Subsequently, amniocentesis was performed and qfPCR with STR markers and karyotyping were carried out. No uniparental disomy in chromosome 7 was identified, while karyotyping result showed normal fetus. Lastly, G-banded chromosome analysis in cord blood confirmed a normal karyotype. Taken together, the results confirmed that confined placental mosaicism (CPM, trisomies 7 and 10) occurred in this case leading to the placental anomalies.

Overall, although an increase of maternal age was observed in the cases with aneuploidies (**Supplementary Figure S3**), no statistical significance was found (Mann-Whitney Test, P=0.115), while no different of paternal age was found.

**Supplementary Figures**


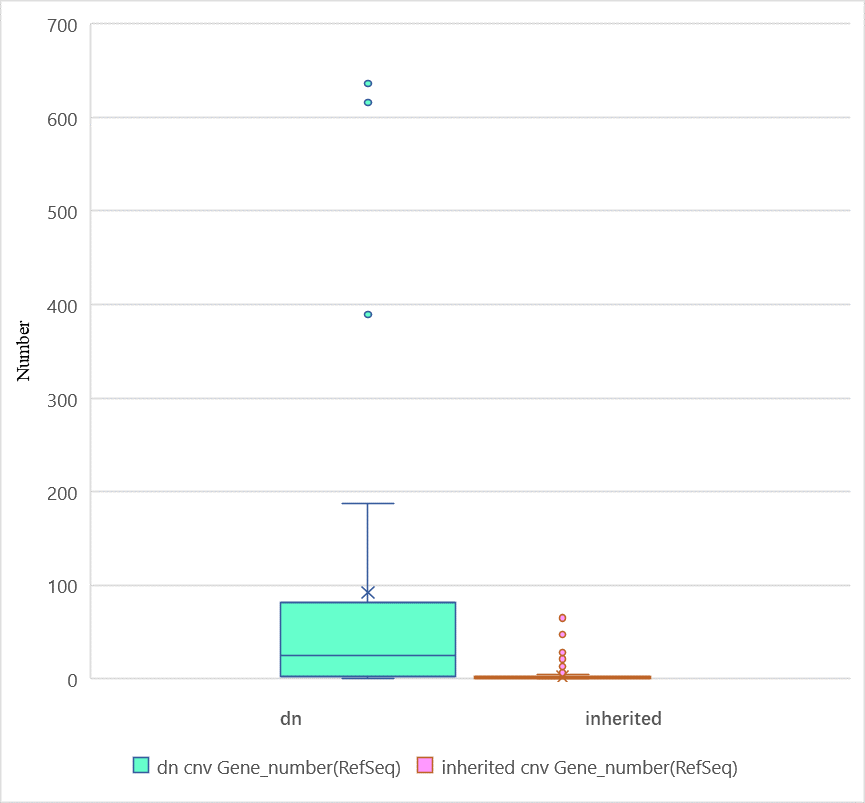


**Supplementary Figure S1. Correlation of the number of genes with the mode of inheritance.** Boxplot of the number of genes (RefSeq) with the CNV’s mode of inheritance. dn refers to *de novo*.


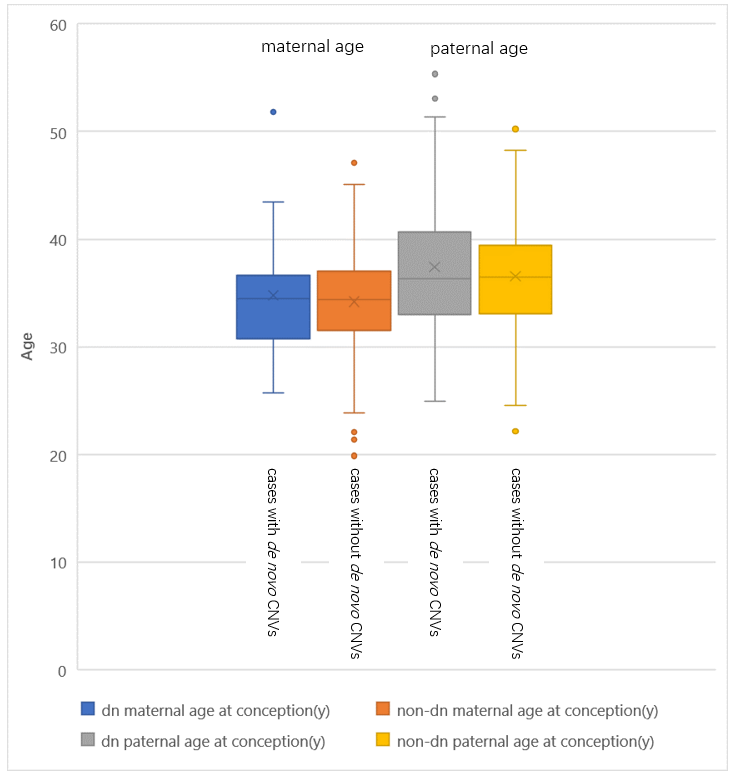


**Supplementary Figure S2. Correlation of parental age with the finding of *de novo* CNVs.** Boxplot of maternal and paternal age between subgroups with or without *de novo* CNVs identified.


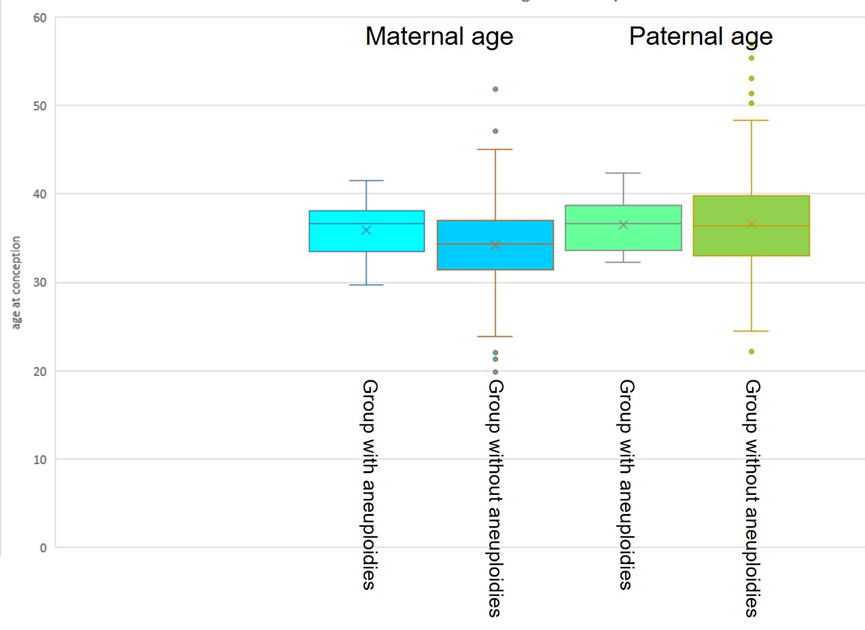


**Supplementary Figure S3. Correlation of parental age with the findings of aneuploidies.** Boxplot of the maternal and paternal age between subgroups with or without aneuploidies identified.

**DATA AVAILABILITY**

Genome sequencing data used in this study have been made available in the CNGB Nucleotide Sequence Archive (CNSA: https://db.cngb.org/cnsa/) under the accession number CNP0002013.
